# Decanoic acid-enriched ketogenic diet in refractory epilepsy

**DOI:** 10.3389/fneur.2025.1524799

**Published:** 2025-01-27

**Authors:** Hui Jin Shin, Seonae Ryu, NaRae Lee, Eunjoo Lee, Ara Ko, Hoon-Chul Kang, Joon Soo Lee, Se Hee Kim, Heung Dong Kim

**Affiliations:** ^1^Division of Pediatric Neurology, Department of Pediatrics, Severance Children’s Hospital, Yonsei University College of Medicine, Seoul, Republic of Korea; ^2^Department of Dietetics, Severance Hospital, Yonsei University College of Medicine, Seoul, Republic of Korea; ^3^Department of Pediatrics, Kangbuk Samsung Hospital, Sungkyunkwan University School of Medicine, Seoul, Republic of Korea

**Keywords:** Decanoic acid, ketogenic diet, epilepsy, refractory, seizure

## Abstract

**Objective:**

To assess the anti-seizure efficacy and safety of a C10-enriched medium-chain triglyceride (MCT) ketogenic diet (KD) compared with the classic KD in pediatric patients with refractory epilepsy.

**Methods:**

This 16-week, open-label, randomized, controlled, crossover pilot study was conducted at Severance Children’s Hospital, Seoul, South Korea, between August 2022 and September 2023. Fifteen pediatric patients with refractory epilepsy were enrolled and received classic KD and C10-enriched KD for 8 weeks each. The study compared seizure reduction rate, tolerability, and safety of the two diets.

**Results:**

Fifteen patients were enrolled. Patients were divided into 2 groups depending on the type of KD initiated. Ten patients completed the trial. Initial treatment with the C10-enriched KD resulted in seizure reduction in all five patients, with two becoming seizure-free. Initial treatment with classic KD was effective in two out of five patients. Upon crossover, those initially on C10-enriched KD maintained their seizure reduction, while patients initially on the classic KD showed additional seizure reduction when switched to C10-enriched KD. Adverse effects included transient hypoglycemia, metabolic acidosis, hypercalciuria, and gastrointestinal symptoms, all of which were manageable.

**Discussion:**

The C10-enriched KD demonstrated comparable efficacy and tolerability to the classic KD, offering a promising option for patients with refractory epilepsy who do not respond adequately to the classic KD alone. This study, the first to directly compare a C10-enriched KD with a classic KD, highlights the potential synergistic effects of decanoic acid.

## 1 Introduction

The addition of medium-chain triglycerides (MCTs) to the ketogenic diet has gained significant scientific attention due to their unique metabolic properties and potential for greater anticonvulsant efficacy compared with traditional long-chain triglycerides (LCTs). Particularly, decanoic acid (C10), which is one of the main components of MCT oil, is recognized for its capacity to optimize mitochondrial functionality, modulate astrocyte activity, increase neuronal GABA production through augmented glutamine supply, and inhibit mTORC1 activity, contributing to improved neurological function (1–3). Decanoic acid also functions as a direct anticonvulsant by attenuating excitatory postsynaptic currents, inhibiting AMPA receptor activity, and promoting GABA synthesis in astrocytes (4, 5).

Previous studies have shown that MCT ketogenic diet (KD) has comparable therapeutic efficacy to the classic KD (6–10). The ratio of MCT incorporated into the KD ranges from 30 to 60% of total energy requirement. Higher ratios of MCT have usually resulted in lower patient compliance with the KD, usually due to poor appetite or gastrointestinal side effects. To overcome these side effects, recently, attempts have been made to improve the compliance of the KD while maintain the therapeutic efficacy. Griffen et al. (11) introduced KetoCal, a novel MCT-enriched liquid nutritional feed, which consisted of a 2.5:1 fat-to-non-fat ratio, with MCT at 25.6% (C10 at 9.8%) of total fat and approximately 19.5% (C10 at 8.2%) of total energy requirement. Beyond just increasing the ratio of MCT, Schoeler et al. (12) introduced K. Vita, a thickened liquid, enriched with decanoic acid (72.1% of total fat, 15–24% of total energy requirement). However, both of these previous studies have not directly compared the therapeutic efficacy of the MCT or C10-enriched KD with the classic KD.

Our group previously demonstrated the therapeutic effects of a liquid ketogenic formula (Classic Ketonia) containing C10 at 0.4% of total fat (13). In this study, we introduce a new liquid ketogenic formula (C10-enriched Ketonia) with a significantly higher concentration of decanoic acid (33.3% of total fat, 28.7% of total energy requirement).

We conducted a pilot study, using an open-label, randomized, controlled, crossover design to evaluate the therapeutic efficacy and safety of a C10-enriched KD, incorporating C10-enriched Ketonia, developed by Namyang Dairy Products. Notably, this study provides a direct comparison between the C10-enriched KD and the classic KD, a comparison not previously undertaken. Our findings highlight the therapeutic potential of a C10-enriched ketogenic diet, supporting the need for further investigation in larger and more diverse epilepsy cohorts.

## 2 Materials and methods

### 2.1 Study design

An open-label, randomized, controlled, crossover design pilot study was conducted between August 2022 and September 2023 at Severance Children’s Hospital, Seoul, South Korea. Patients were enrolled to receive either the classic KD for 8 weeks followed by the C10-enriched KD for another 8 weeks, or vice versa, totaling 16 weeks. Prior to study commencement, we obtained complete approval from the Institutional Review Board of Severance Hospital, Yonsei University College of Medicine (4–2022-0758). Written consent was provided by patients and guardians.

### 2.2 Screening of eligible patients

Patients underwent initial screening for eligibility. Clinical information and other test results including epilepsy diagnoses, seizure semiology, previous electroencephalogram (EEG) and brain magnetic resonance imaging (MRI) were obtained. The inclusion criteria were as follows: (1) age between 3 months and 20 years, (2) diagnosed with refractory epilepsy, having persistent seizures even after administration of two or more ASMs, (3) history of seizures within 2 weeks before the study, (4) baseline seizure frequency of more than 2 times per month Patients with a broad spectrum of seizure frequency was included to reflect the clinical heterogeneity of refractory epilepsy. The exclusion criteria were as follows: (1) admitted for medical issues other than epilepsy, (2) pregnancy or planning to become pregnant, (3) history of milk allergy or lactose intolerance, (4) contraindications to KD such as diagnosed metabolic diseases, (5) diagnosis of a progressive neurological disorder. Patients with a prior history of KD were eligible for inclusion if their last KD intervention occurred at least 1 year before study enrollment.

Prior to randomization into treatment groups, patients completed a 2-week observation period to confirm eligibility for study inclusion. Following random allocation, patients commenced either the classic KD or the C10-enriched KD for 8 weeks. Thereafter, they switched to the other regimen for another 8 weeks, resulting in a trial duration of 16 weeks. For both diet regimens, if the patient could not tolerate the baseline KD with a 3:1 ratio of fat to non-fat, the ratio was adjusted to the modified Atkins diet (MAD) within 1 week (Supplementary Figure S1). Patients visited the clinic for physical examination, clinical evaluation, and biochemical tests via blood and urine samples at 4, 8, and 16 weeks of follow-up.

### 2.3 Ketogenic diet

Ketogenic diet regimens adhered to a revised version of the Johns Hopkins Protocol, as modified by Severance Hospital (14–16). Baseline KD regimens included the classic ketogenic diet (KD) with a 3:1 fat-to-nonfat ratio (protein and carbohydrate) and the modified Atkins diet (MAD) with a 1.7:1 ratio. These regimens were supplemented with permitted foods consistent with KD, including beef, cheese, vegetable oils, and almonds. Classic Ketonia was added to classic KD, while C10-enriched Ketonia, distinguished by its high decanoic acid content, was included in the C10-enriched KD. The classic KD was administered using Classic Ketonia, while the C10-enriched KD utilized C10-enriched Ketonia, (Supplementary Table S1).

Energy targets for each patient on all KDs were calculated based on age, height, and body weight. Initial calorie prescription was 75% of the basal energy expenditure for age. Without an initial fasting period, on the first day of each new KD, patients received meals amounting to one-third of the planned daily energy requirement; on the second day, it was two-thirds of the daily energy requirement; and from the third day onwards, patients received their full energy requirements.

Both Classic and C10-enriched Ketonia were liquid ketogenic formulations produced by Namyang Dairy Products (Seoul, South Korea), with patients consuming one 180 mL pack three times daily. Classic Ketonia, previously introduced at our center, contains refined olive oil (extra olive oil, mixed d-tocopherol concentrate), soybean oil, whey protein concentrate, sodium caseinate, and PE-30A (an emulsion stabilizer) (13). The Classic Ketonia formulation provided 0.05 g/100 mL of decanoic acid. In contrast, the C10-enriched Ketonia was identical in composition except for an increased decanoic acid content of 4.0 g/100 mL (Supplementary Table S2). Overall, the classic KD included MCT at 4.2% of total fat and decanoic acid at 0.4% of total fat, while the C10-enriched KD consisted of MCT at 83.3% of total fat and decanoic acid at 33.3% of total fat (Table 1). Each patient also received sugar-free L-carnitine, elemental calcium, vitamin D2, multivitamins, and mineral supplements to prevent potential micronutrient deficiencies.

**Table 1:** Total fat composition for each diet.

Type of fat	Composition (%)	
	Classic KD	C10-enriched KD
SCT (C4–C6)	0.04	0.04
MCT (C8–C12)	4.2	83.3
C8	0.6	50
C10	0.4	33.3
LCT (C14–C22)	95.8	16.7
Total	100	100

C10, decanoic acid; KD, ketogenic diet; LCT, long-chain triglyceride; MCT, medium-chain triglyceride.

## 3 Results

### 3.1 Patient demographics and clinical characteristics

**Figure 1:**
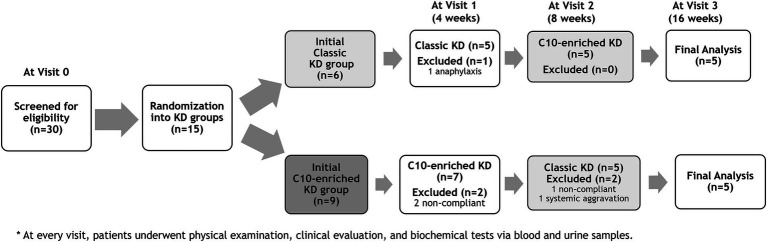
Study design and patient flow chart: Classic KD vs C10-enriched KD.

Between August 2022 and September 2023, fifteen patients were enrolled, with six patients initially assigned to the classic KD group and nine to the C10-enriched KD group. Five patients withdrew early from the trial and were subsequently excluded from the analysis. Four patients discontinued the study due to challenges in adhering to the KD protocol. One patient experienced a mild anaphylactic reaction, including brief urticaria, tachypnea, and tachycardia, following the initiation of the C10-enriched ketogenic diet on day one. These symptoms were likely related to an undisclosed sensitivity to milk and soy-based products, prompting the patient to withdraw from the study as a precaution (Figure 1).

The mean age at the start of the study was 8.9 years. Etiology of epilepsy varied among patients, including seven patients (7/15, 47%) with unknown etiology, three (20%) with structural and infection etiologies, and two (13%) with genetic etiologies. Eight patients (8/15, 53%) were diagnosed with non-lesional focal epilepsy, and six (6/15, 40%) with developmental and epileptic encephalopathy. Majority of patients (12/15, 80%) exhibited generalized seizures. Eleven (11/15, 73%) patients demonstrated focal epileptiform discharges on EEG. Four patients had abnormal brain MRI findings such as perisylvian polymicrogyria and cortical dysplasia. The enrolled patients were administered with an average number of five ASM at baseline. Four out of 15 patients (27%) had a prior history of KD, with three patients on classic KD and one patient on MAD. Previously, these patients discontinued KD due to either poor compliance or ineffectiveness of KD (Table 2).

**Table 2:** Demographics and clinical characteristics of patients.

Variable	*n* = 15
Sex (male)	8 (53%)
Age at ketogenic diet	8.9 ± 4.6 (5.8–12.9)
Age at seizure onset (yr)	2.9 ± 3.7 (0.2–4.9)
Seizure type	
Generalized	12 (80%)
Focal	3 (20%)
Seizure frequency	
Daily	9 (60%)
Weekly	3 (20%)
Monthly	3 (20%)
Etiology	
Structural	3 (20%)
Infection	2 (13%)
Genetic	3 (20%)
Unknown	7 (47%)
Epilepsy type	
Lesional focal epilepsy	1 (7%)
Non-lesional focal epilepsy	8 (53%)
DEE	6 (40%)
EEG	
Generalized	4 (27%)
Focal	11 (73%)
Abnormal MRI	4 (27%)
Number of ASM at baseline	5.13 ± 1.9 (4.0–6.0)
Previous history of KD	4 (27%)
KD 3:1	3 (20%)
MAD	1 (7%)
Reason for withdrawal of prior KD	
Poor adherence to diet	2 (13%)
Ineffective	2 (13%)
Previous history of epilepsy surgery	4 (27%)
Developmental delay	12 (80%)
Mobility status	
Independent walking	12 (80%)
Walking with support	1 (7%)
Bed-ridden	2 (13%)

ASM, anti-seizure medication; EEG, electroencephalogram; KD, ketogenic diet; MAD, modified Atkins diet; MRI, magnetic resonance imaging.

### 3.2 Treatment outcome

**Figure 2:**
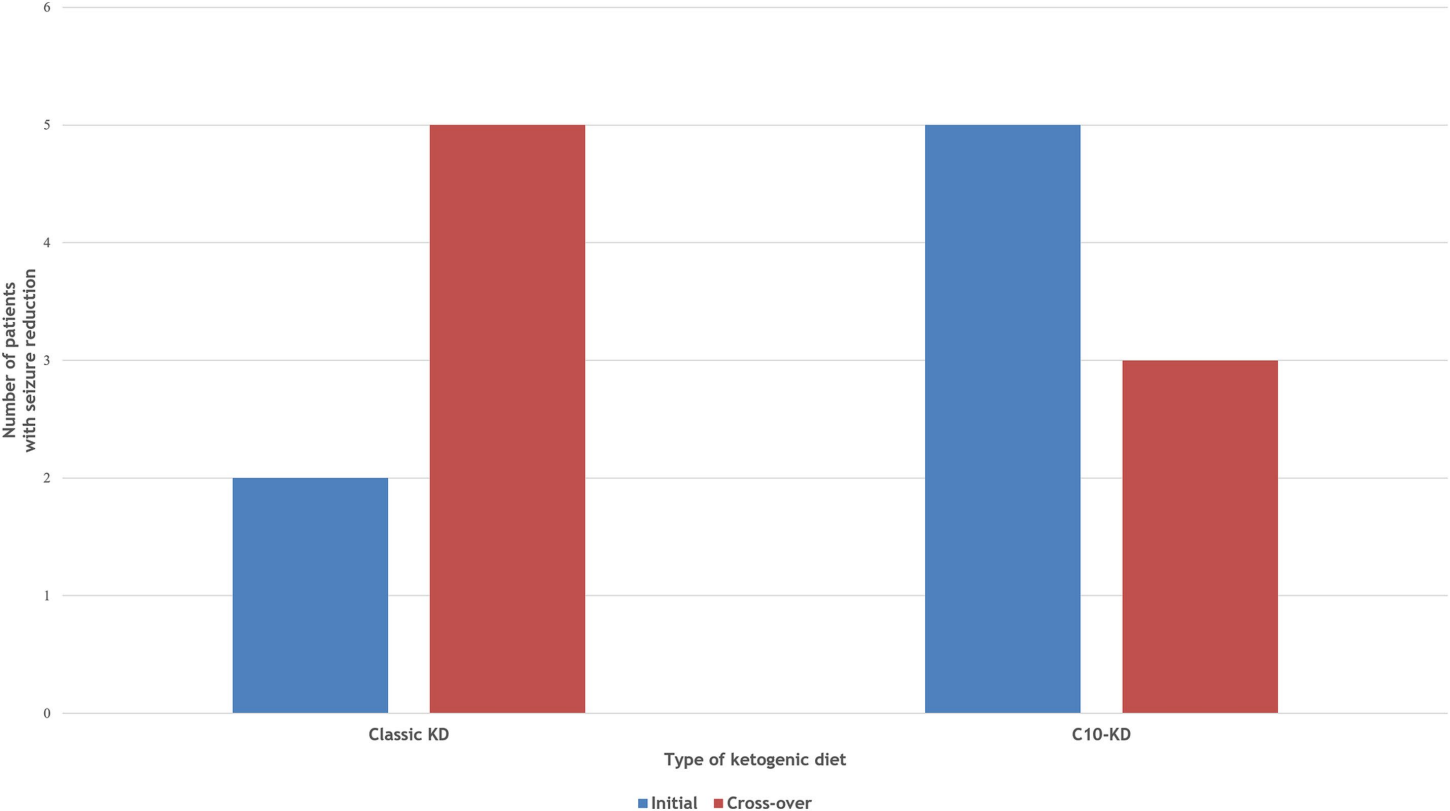
Comparison of therapeutic efficacy between Classic KD vs C10-enriched KD. Two patients achieved ≥50% seizure reduction on the initial classic KD, increasing to three after cross-over to C10-KD. All patients responded to the initial C10-KD, maintaining seizure reduction after cross-over to classic KD.

Ten out of fifteen patients (67%) completed the 16-week trial. One patient (P4) switched from the classic KD regimen of a 3:1 ratio to the MAD during the first week due to repeated hypoglycemic events. Initial treatment with the C10-enriched KD resulted in seizure reduction in all five patients, with two (P6, P8) achieving complete seizure freedom, whereas the classic KD was effective in only two out of five patients (40%). Upon crossover, patients who started on the C10-enriched KD maintained their seizure reduction, while those switching from the classic KD to the C10-enriched KD experienced additional benefits, with 60% (three out of five) showing further seizure reduction (Figure 1). Notably, two of the three patients who did not respond well to the classic KD responded favorably to the C10-enriched KD, including one with a marked reduction in focal seizures. Only one patient (P4) who initially benefited from the classic KD did not maintain similar improvements on the C10-enriched KD, primarily due to lower adherence toward the trial’s end (Table 3). These findings indicate that the C10-enriched KD may provide an added benefit in seizure control, particularly for patients with limited response to the classic KD (Figure 2).

**Table 3:** Clinical characteristics and seizure outcomes for each patient who completed the 16-week study on the ketogenic diet.

	Initial classic ketogenic diet group					Initial C10-enriched ketogenic diet group				
	P1	P2	P3	P4	P5	P6	P7	P8	P9	P10
Sex, Age	M, 17 y	M, 15 y	M, 12 y	F, 9 y	F, 7 y	F, 13 y	M, 5 y	M, 5 y	M, 3 y	F, 1 y 6 m
Age at seizure onset	12 y 6 m	8 y	1 y 6 m	5 y	1 d	6 y	5 m	6 m	2 m	3 m
Seizure type	Generalized, tonic, Tonic–clonic	Focal, Tonic–clonic	Generalized, Atonic	Focal, impaired awareness followed by tonic	Generalized, Tonic–clonic	Generalized, Tonic–clonic	Generalized, Tonic	Generalized, Tonic	Focal, myoclonic	Generalized, spasm
Etiology	Unknown	Infection (Encephalitis, unknown)	Infection (Encephalitis, viral)	Unknown	Structural (Polymicrogyria)	Unknown	Structural (Cortical dysplasia)	Genetic (15q11.2 duplication)	Unknown	Genetic (CDKL5)
Epilepsy type	Non-lesional focal	DEE	DEE	Non-lesional focal	Non-lesional focal	Non-lesional focal	Lesional focal	Non-lesional focal	DEE	DEE
EEG	Focal, occasional ED	Focal, occasional ED	Generalized, frequent ED	Focal, rare ED	Focal, frequent ED	Focal, slowing only	Focal, very frequent ED	Focal, frequent ED	Focal, very frequent	Generalized, occasional ED
MRI	Normal	Normal	Normal	Normal	Perisylvian polymicrogyria	Normal	Rt. Frontal Cortical dysplasia	Normal	Normal	Normal
Number of ASM at baseline	4	5	5	4	5	3	4	3	5	5
Previous history of KD, last KD date, reason for withdrawal	None	None	None	None	None	None	KD 3:1, for 3 mo, 2 yrs. ago, low adherence	None	KD 3:1, for 6 mo, 2 yrs. ago, ineffective	MAD, for 3 mo, 1 yr. ago, ineffective
Previous history of epilepsy surgery	None	VNS	None	None	None	None	Resective surgery	None	Resective surgery	None
Developmental delay, mobility status	Yes, independent walking	Yes, independent walking	Yes, independent walking	Yes, independent walking	Yes, independent walking	No, independent walking	Yes, independent walking	Yes, walk with support	Yes, independent walking	Yes, bed-ridden
Seizure frequency: At baseline	1/week	5/week	3–4/day	2–3/month	5/week	2/month	30–50/day	2–3/day	30/day	7/day
After 2 months of classic KD	1/month	5/week	3–4/day	None^***^	3-4/week	None	5–8/day	None	4/week	2-3/day
After 2 months of C10 KD	1/2 months	5/week	0–5/day	2/month^*^	5/week^*^	None	4–5/day	None	2/week	3/day
Classic KD effective?	Y, initial	N, initial	N, initial	Y, initial	N, initial	Y, crossover	Y, crossover	Y, crossover	Y, crossover	Y, crossover
C10-enriched KD effective?	Y, crossover	N, crossover	Y, crossover	N^*^, crossover	Y^**^, crossover	Y, initial	Y, initial	Y, initial	Y, initial	Y, initial
Side effects of KD	GI Symptoms with C10-enriched KD	Transient metabolic acidosis, C10-enriched KD	GI Symptoms with C10-enriched KD, Hypercalciuria with classic KD	Transient hypoglycemia, metabolic acidosis with C10-enriched KD	None	None	None	None	None	None

* reported poor compliance to C10 KD ** reported significant decrease in seizure intensity (shorter duration in seizure and decreased tonicity). *** changed from a KD 3:1 ratio to MAD during 1st week. DEE, developmental and epileptic encephalopathy; cKD, classic ketogenic diet; C10KD, C10-enriched ketogenic diet; GI, gastrointestinal; ASM, anti-seizure medication; EEG, electroencephalogram; MRI, magnetic resonance imaging; MAD, modified Atkins diet.

The average age of patients who responded well to KD (P1, P6–10) was 7.6 years, younger than the 10.9 years observed in non-responders (P2–P5). No significant changes were observed in anthropometric parameters such as body weight, height, or body mass index among patients during the trial (Supplementary Table S3). Additionally, no significant changes were observed in biochemical laboratory tests conducted throughout the study.

### 3.3 Adverse effects

Adverse effects of the C10-enriched KD included transient hypoglycemia and metabolic acidosis in two patients (P2, P4), transient hypercalciuria in one patient (P3), and transient gastrointestinal symptoms in two patients (P1, P3). For hypoglycemia, intravenous dextrose injection was administered when glucose levels were < 40 in asymptomatic patients and < 50 in symptomatic patients until resolution, typically within the first 3–5 days of the diet. For metabolic acidosis, patients were administered intravenous and oral sodium bicarbonate until resolution, typically within the first 2 weeks into the diet. Patient P3 recovered from hypercalciuria without further intervention. Transient gastrointestinal symptoms were resolved within the first to second week of the diet with or without oral gastrointestinal medication such as domperidone maleate or magnesium hydroxide (Table 3).

## 4 Discussion

The effect of decanoic acid within KD treatment for epilepsy remains inadequately explored in human studies. Our 16-week prospective pilot study focused exclusively on a pediatric cohort with refractory epilepsy of various etiologies, comparing the efficacy and tolerability of a C10-enriched KD to that of a classic KD. We administered C10-enriched Ketonia, which contained MCTs (83.3% of total fat) and C10 (33.3% of total fat), in conjunction with KD to explore potential synergistic effects. Our findings suggest that the efficacy and tolerability of the C10-enriched KD are comparable to those of the classic KD. The majority of enrolled patients showed a ≥ 50% reduction in seizures when treated with C10-enriched KD. The banana-flavored C10-enriched Ketonia was also well-accepted by the pediatric patients. Adverse effects were primarily mild gastrointestinal disturbances, similar to those in other KD studies.

Griffen et al. (11) conducted a two-month study introducing KetoCal, an MCT-enriched liquid feed, to patients with refractory epilepsy on a KD. KetoCal had a 2.5:1 fat-to-non-fat ratio, with MCTs constituting 25.6% of total fat and C10 at 9.8%. The study included both children and adults, with a control period where patients were on KD only, followed by an intervention period where KetoCal was introduced. Both children and adults using KetoCal exhibited improved KD adherence without gastrointestinal side effects, maintaining therapeutic efficacy comparable to the control group. Although not particularly enriched in decanoic acid, KetoCal showed potential in reducing seizures (17).

Schoeler et al. (12) conducted a 12-week study introducing K. Vita, a decanoic acid-enriched medical food, to children with Dravet syndrome or early-onset genetic epilepsy and adults with refractory epilepsy. K. Vita contained 98.5% MCTs, with C10 constituting 72.1% of total fat. Participants followed a diet excluding high-refined sugar foods and beverages. In their trial, six of 16 children (38%) and eight of 16 adults (50%) achieved a ≥ 50% reduction in seizures or paroxysmal events. K. Vita was generally well-accepted by patients. Gastrointestinal disturbances were the primary adverse effects which decreased over time (12).

Unlike the two previously mentioned studies, our pilot study directly compared the therapeutic outcomes of a standard KD with a C10-enriched KD. Our findings indicate that the C10-enriched KD provides comparable efficacy in seizure reduction, suggesting it as a promising alternative for pediatric patients with refractory epilepsy who do not achieve adequate seizure control with the classic KD alone. These results align with the observations of Schoeler et al. (12, 20) and Griffen et al. (11), underscoring the potential synergistic benefits when both dietary approaches are integrated.

While both decanoic and octanoic acid levels increase during an MCT KD, decanoic acid exhibits notable efficacy in seizure management, likely due to its brain-sparing properties via beta-oxidation (12, 18–20). Decanoic acid undergoes limited beta-oxidation in the liver, leading to its accumulation in the bloodstream and subsequent transport to the brain. Once in the brain, decanoic acid acts as a direct anticonvulsant by inhibiting excitatory postsynaptic currents and AMPA receptors and increase in GABA synthesis in astrocytes. (21–24) Meanwhile, octanoic acid undergoes more extensive beta-oxidation in the liver, resulting in lower brain levels compared to decanoic acid.

Chang et al. (25) conducted both *in vitro* and *in vivo* studies to compare the effects of various medium-chain fatty acids on seizure control. In their in vitro pentylenetetrazol model, octanoic acid had no significant effect on seizure control while decanoic acid exhibited a marked reduction in epileptiform discharges. Additionally, in vivo studies using a drug-resistant status epilepticus model demonstrated that decanoic acid was more potent in controlling seizures compared to valproic acid and showed less sedation and enhanced neuroprotection (25). Tan et al. (26) also showed that that chronic feeding of tridecanoin (a triglyceride form of decanoic acid), but not trioctanoin (a triglyceride form of octanoic acid), was reproducibly anticonvulsant in mouse seizure models. Decanoic acid increased mitochondrial function and antioxidant capacity, leading to better seizure control compared to octanoic acid (26).

Decanoic acid also acts as an agonist of peroxisome proliferator-activated receptor *γ* (PPARγ), promoting mitochondrial biogenesis and enhancing mitochondrial complex I activity (1, 2). This improvement in mitochondrial function is shown to synergistically enhance the seizure-reducing effects of the KD in mouse models (27). Additionally, Warren et al. (3) demonstrated that decanoic acid decreases mTORC1 activity under low insulin and glucose conditions, similar to those induced by KD, thereby reducing neuronal excitability. These findings support our study, suggesting that decanoic acid may enhance the efficacy of the ketogenic diet in reducing seizure activity among patients (3, 28).

In our study, younger patients tended to be more responsive to KD than older patients. Heales et al. (29) also demonstrated that younger patients exhibit higher rates of glycolysis and beta-oxidation, alongside increased concentrations of C10, than older patients (29). Although we cannot deduce such conclusions from out study, it may be noteworthy to further investigate the effect of age in the therapeutic efficacy of decanoic acid-enriched KD across different age groups.

Limitations of this study include the following: (1) the number of patients was limited, (2) plasma levels of MCTs such as C10 and C8 were not measured to correlate with therapeutic efficacy, (3) an absence of further investigation on the cognitive/behavioral impact of the KD. To address these limitations, it is essential to conduct a more extensive clinical trial, incorporating meticulous monitoring of laboratory parameters and clinical developmental scales.

Our study demonstrated that a C10-enriched KD is comparable to the classic KD in treating refractory epilepsy. Decanoic acid shows promise for incorporation into KD regimens, offering comparable efficacy and tolerability. This approach could potentially ease the strict dietary restrictions associated with traditional KDs while maintaining therapeutic benefits for patients with refractory epilepsy. Further research is warranted to explore strategies to improve adherence to KDs and enhance their clinical outcomes.

## Data Availability

The original contributions presented in the study are included in the article/Supplementary material, further inquiries can be directed to the corresponding authors.
