# Supplementary material for: Decanoic acid-enriched ketogenic diet in refractory epilepsy
Source: Front Neurol. 2025 Jan 27;16:1524799. doi: 10.3389/fneur.2025.1524799 (PMC11809036; doi:10.3389/fneur.2025.1524799)
Supplement: Supplementary file 1 [file Table_1.docx]

Supplementary Material

# Supplementary Figures and Tables

## Supplementary Figures

**Supplementary Figure S1.** **Study Timeline**

* At every visit, patients underwent physical examination, clinical evaluation, and biochemical tests via blood and urine samples.

## Supplementary Tables

**Supplementary Table S1. Nutritional composition of both Ketonias**

| **Nutritional composition** | **Composition (%)** | | | |
| --- | --- | --- | --- | --- |
|  | **KD 3:1** | | **MAD** | |
|  | **Classic** | **C10-enriched** | **Classic** | **C10-enriched** |
| **Fat** | 87 | 87 | 79 | 79 |
| SCT | 0.09 | 0.09 | 0.08 | 0.7 |
| MCT | 3.6 | 74.9 | 3.2 | 68.2 |
| C8 | 0.022 | 37.8 | 0.019 | 39.5 |
| C10 | 0.014 | 25.3 | 0.013 | 23.1 |
| LCT | 83.3 | 11.3 | 13 | 13 |
| **Protein** | 8 | 8 | 15 | 15 |
| **Carbohydrate** | 5 | 5 | 6 | 6 |
| Total | 100 |  | 100 | 100 |

C10; decanoic acid; KD, ketogenic diet; LCT, long-chain triglyceride; MAD, modified Atkins diet; MCT, medium-chain triglyceride; SCT, short-chain triglyceride.

**Table S2. Composition of energy requirements**

| **Nutritional composition (unit)** | **Classic Ketonia**  **(per 100 mL)** | **C10-enriched Ketonia**  **(per 100 mL)** |
| --- | --- | --- |
| **Energy (Kcal)** | **120** | **120** |
| **Sodium (mg)** | **50** | **50** |
| **Carbohydrate (g)** | **1** | **1** |
| **Sugar (g)** | **0** | **0** |
| **Fat (g)** | **12** | **12** |
| **Trans Fat (g)** | **0** | **0** |
| **Saturated Fat (g)** | **2.5** | **11** |
| **LCT** | **11.5 (95.8% of total fat)** | **2.0 (16.7% of total fat)** |
| **MCT** | **0.50 (4.2%)** | **10.0 (83.3%)** |
| **Octanoic acid (C8:0)** | **0.07 (0.6%)** | **6.0 (50%)** |
| **Decanoic acid (C10:0) (g)** | **0.05 (0.4%)** | **4.0 (33.3%)** |
| **C12 (g)** | **0.38 (3.2%)** | **0.010 (0.08%)** |
| Cholesterol (mg) | 4 | 4 |
| **Protein (g)** | **2** | **2** |
| Vitamin A (μgRE) | 75 | 75 |
| Vitamin B1 (mg) | 0.13 | 0.13 |
| Vitamin B2 (mg) | 0.13 | 0.13 |
| Vitamin B6 (mg) | 0.13 | 0.13 |
| Vitamin B12 (μg) | 0.4 | 0.4 |
| Vitamin C (mg) | 12 | 12 |
| Vitamin D (μg) | 1.2 | 1.2 |
| Vitamin E (mgα-TE) | 1 | 1 |
| Vitamin K1 (μg) | 7 | 7 |
| Pantothenic acid (mg) | 0.6 | 0.6 |
| Niacin (mgNE) | 1.2 | 1.2 |
| Folic acid (μg) | 27 | 27 |
| Biotin (μg) | 3.6 | 3.6 |
| Calcium (mg) | 120 | 120 |
| Phosphorus (mg) | 72 | 72 |
| Magnesium (mg) | 9.6 | 9.6 |
| Potassium (mg) | 102 | 102 |
| Iron (mg) | 1.9 | 1.9 |
| Zinc (mg) | 1.2 | 1.2 |
| Manganese (μg) | 7.2 | 7.2 |
| Copper (μg) | 78 | 78 |
| Iodide (μg) | 14.4 | 14.4 |
| Inositol (mg) | 4.6 | 4.6 |
| Chloride (mg) | 72 | 72 |
| Choline (mg) | 9.6 | 9.6 |
| L-carnitine (mg) | 5 | 5 |

LCT, long-chain triglyceride; MCT, medium-chain triglyceride.

**Table S3. Anthropometrics before and after ketogenic diet intervention**

| Patient | Initial body weight (kg) | Initial BMI (kg/m^2^) | Body weight change during cKD (kg) | BMI change during cKD (kg/m^2^) | Body weight change during C10KD (kg) | BMI change during C10KD (kg/m^2^) |
| --- | --- | --- | --- | --- | --- | --- |
| 1 | 52 | 16 | -1.4 | -0.48 | -4 | -1.19 |
| 2 | 61 | 23 | -1.9 | -0.61 | 0.9 | 0.15 |
| 3 | 35 | 18 | -3 | -2.47 | -2.7 | -1.97 |
| 4 | 42 | 22 | -6.3 | -3.44 | 0 | 0 |
| 5 | 25 | 17 | -0.5 | -0.79 | -1 | -0.36 |
| 6 | 66 | 25 | -2.5 | -0.9 | -7.7 | -3.02 |
| 7 | 26 | 21 | -0.9 | -0.8 | -0.6 | -1.73 |
| 8 | 17 | 17 | 0.7 | 1.36 | -0.5 | -1.56 |
| 9 | 16 | 16 | 1 | 0.3 | 2 | 2.57 |
| 10 | 10 | 16 | -0.6 | -1.81 | -1.2 | -3.54 |
| 11 | 20 | 14 |  |  |  |  |
| 12 | 20 | 15 |  |  |  |  |
| 13 | 35 | 22 |  |  |  |  |
| 14 | 18 | 11 |  |  |  |  |
| 15 | 22 | 17 |  |  |  |  |

cKD, classic ketogenic diet; C10KD, C10-enriched ketogenic diet.
